# Improving Antigenic Peptide Vaccines for Cancer Immunotherapy Using a Dominant Tumor-specific T Cell Receptor*

**DOI:** 10.1074/jbc.M113.509554

**Published:** 2013-10-08

**Authors:** Jonathan D. Buhrman, Kimberly R. Jordan, Daniel J. Munson, Brandon L. Moore, John W. Kappler, Jill E. Slansky

**Affiliations:** From the ‡ Integrated Department of Immunology and; the § Department of Surgery, University of Colorado School of Medicine, Aurora, Colorado 80045 and; the ¶ Howard Hughes Medical Institute at National Jewish Health, Denver, Colorado 80206

**Keywords:** Antigen, Peptide Arrays, T Cell, T Cell Receptor, Tumor Immunology, Vaccines

## Abstract

Vaccines that incorporate peptide mimics of tumor antigens, or mimotope vaccines, are commonly used in cancer immunotherapy and function by eliciting increased numbers of T cells that cross-react with the native tumor antigen. Unfortunately, they often elicit T cells that do not cross-react with or that have low affinity for the tumor antigen. Using a high affinity tumor-specific T cell clone, we identified a panel of mimotope vaccines for the dominant peptide antigen from a mouse colon tumor that elicits a range of tumor protection following vaccination. The TCR from this high affinity T cell clone was rarely identified in *ex vivo* evaluation of tumor-specific T cells elicited by mimotope vaccination. Conversely, a low affinity clone found in the tumor and following immunization was frequently identified. Using peptide libraries, we determined if this frequently identified TCR improved the discovery of efficacious mimotopes. We demonstrated that the representative TCR identified more protective mimotopes than the high affinity TCR. These results suggest that targeting a dominant fraction of tumor-specific T cells generates potent immunity and that consideration of the available T cell repertoire is necessary for targeted T cell therapy. These results have important implications when optimizing mimotope vaccines for cancer immunotherapy.

## Teaser

**Background:** Vaccination with mimotopes, peptide mimics of epitopes, stimulates a range of T cell protection.

**Results:** Mimotopes identified from peptide libraries by T cells with common receptors increased immunity more than those with rare high affinity receptors.

**Conclusion:** T cell prevalence must be considered when designing peptide vaccines.

**Significance:** Optimizing mimotopes will improve antigen-specific vaccines for applications including cancer immunotherapies.

## Introduction

Cytotoxic T lymphocytes express T cell receptors (TCRs)^3^ that are generated by gene rearrangements and random nucleotide additions during T cell development (1, 2). The most highly variable region of the TCR, the complementarity-determining region 3 (CDR3), confers the specificity of TCRs by interacting with short peptide sequences presented on the cell surface in the peptide binding groove of MHC class I molecules. Although TCRs are exquisitely specific, they are also flexible and can adopt different conformations, depending on the peptide-MHC (pMHC) complex (3, 4). One recent report suggests that a single TCR can recognize more than a million different peptides (5). TCR molecules can also adopt different binding orientations to recognize the same pMHC complex (6–8), further contributing to TCR degeneracy. The flexibility and cross-reactivity of TCRs is essential for the host to overcome the physical constraints on the T cell repertoire and the limited number of potential peptide antigens that can be presented by host MHC (9, 10). However, T cell cross-reactivity may also contribute to the initiation of autoimmune responses (11, 12).

Autoreactive cytotoxic T lymphocytes with high affinity for autoantigens are usually deleted during development in the thymus. However, T cells specific for self-antigens can escape central tolerance and enter the periphery, where they mediate autoimmune disease and respond to self-tumor antigens (13). Some autoreactive T cells escape central tolerance because they have a low affinity for self-antigens, below the threshold for negative selection (14–16). Peripheral tolerance mechanisms, such as anergy and ignorance, normally suppress the expansion of these cells (17). However, vaccination with foreign or altered self-antigens that cross-react with these low affinity T cells overcomes peripheral tolerance, leading to their proliferation and differentiation (18, 19). Overcoming tolerance is particularly relevant toward the development of vaccines against cancers that express tumor-associated self-antigens.

T cell tolerance, low affinity of self-antigens for MHCs or TCRs, and the immunosuppressive environment of tumors may all contribute to the minimal expansion of tumor-specific T cells in response to peptide vaccines used to treat cancer patients (20). Mimotopes, also referred to as altered peptide ligands or heteroclitic peptides, are peptides with amino acid substitutions within the native tumor antigen that potentially improve immunogenicity by exploiting T cell cross-reactivity (21–23). Determining which mimotopes are most effective upon vaccination and how best to identify those mimotopes remains a challenge. Understanding the fundamental requirements for the activation and expansion of tumor-specific T cells may facilitate the discovery of ideal peptide vaccines for use in a clinical setting.

Targeting specific T cell clonotypes, especially those associated with enhanced T cell function, is one strategy to develop more effective mimotope vaccines (9, 24, 25). However, vaccination with mimotopes unpredictably affects the responding T cell clonotypes (26, 27) and often results in the expansion of T cells with lower affinity for native tumor antigens (28–30). Numerous mimotopes have been identified or screened using T cell clones with high affinity for the native tumor antigen expanded *in vitro* (31–37). Whether mimotopes identified by these T cell clones elicit the same high affinity TCR clonotypes after vaccination remains unclear.

Using the mouse colon carcinoma CT26, we have applied several screening techniques for peptide mimics of the immunodominant self-antigen gp70_432-431_ (AH1), including positional scanning formats (37), combinatorial peptide libraries (36), and baculovirus-encoded peptide libraries (38). We screened these peptide libraries for candidate mimotope vaccines based on the response of a high affinity tumor-specific T cell clone, CT, which was propagated after limiting dilution of T cells from a CT26-GM-vaccinated mouse (37). Although vaccination with the candidate mimotopes elicited more AH1-tetramer-specific T cells than vaccination with the AH1 peptide itself, not all mimotopes significantly improved anti-tumor immunity (39). To understand the range of anti-tumor immunity elicited by mimotopes, we sequenced the tumor-specific TCRs responding to different mimotope vaccines (40). These studies revealed a frequently expressed motif within the CDR3 β-chain in mice vaccinated with more protective mimotopes. Therefore, we expanded the 1D4 T cell clone, which expressed a common CDR3β motif, bound to mimotopes that prevented tumor growth, and did not bind to the less protective mimotopes (40).

We hypothesized that screening mimotope libraries with TCRs that are representative of endogenous tumor-specific T cells, rather than using rare high affinity clones, would improve the discovery of efficacious mimotopes for cancer immunotherapy. We demonstrate here that the 1D4 TCR identifies more protective mimotopes and, perhaps more importantly, fewer poorly protective mimotopes than the CT TCR despite the 1D4 TCR having a lower affinity for the AH1 peptide. Screening a recombinant baculovirus peptide-MHC library with the 1D4 TCR, we identified candidate mimotopes that enhanced the expansion of AH1-specific T cells compared with those identified by the CT TCR. Furthermore, T cells elicited by 1D4-identified mimotopes had increased functional recognition for the native AH1 tumor antigen. These results have important implications for developing strategies to identify effective peptide vaccines for immunotherapy. Recent advances in sequencing technology allowed for in depth investigation of endogenous T cell responses within tumors and the identification of optimal TCRs to be exploited for mimotope discovery.

## EXPERIMENTAL PROCEDURES

### 

#### 

##### Mice

6–8-week-old female BALB/cAnNCr mice were purchased from the National Cancer Institute/Charles River Laboratories. All animal protocols were reviewed and approved by the Institutional Animal Care and Use Committee at National Jewish Health.

##### Peptides

Peptide sequences used but not listed in Table 3 are β-gal (TPHPARIGL), AH1 (SPSYVYHQF), and the mimotopes of AH1 (amino acid substitutions are underlined): A5 (SPSYAYHQF), F1A5 (FPSYAYHQF), WMF (SPTY*A*YWMF), A5A7 (SPSYAYAQF), and A5Y7 (SPSYAYYQF). Soluble synthetic peptides were ≥95% pure (Chi Scientific).

##### Recombinant Baculoviruses Expressing pMHC and TCR Molecules

Recombinant baculoviruses (rBVs) were engineered to express a mouse MHC class I molecule, H-2L^d^, using a modified version of the pAcUW31 vector, referred to here as pBACpHp10 (41). Sequences encoding the H-2L^d^ molecule, as well as the indicated peptide covalently linked to mouse β_2_-microglobulin, were inserted downstream of the pH and p10 promoters, respectively (42). Peptides were covalently linked to the mouse β_2_-microglobulin via a glycine/serine-rich linker attached to the C terminus of the peptide.

TCR α- and β-chains were inserted into the pBACp10pH vector downstream of the p10 and pH promoters, respectively. We generated the CT TCR in BVs as previously described (37, 38). The 1D4 TCR was isolated from an AH1-specific T cell clone from the spleen of an immunized BALB/c mouse (40). mRNA was isolated from ∼1 × 10^5^ cells using the RNeasy Minikit (Qiagen), and cDNA was synthesized using the Quantitect reverse transcriptase kit (Qiagen) according to the manufacturer's instructions. Standard cloning techniques were used to insert the TCR α- and β-chain into the pBACp10pH vector. Plasmid DNA from pBACp10pH vectors was co-transfected by calcium phosphate transfection with Sapphire^TM^ BV DNA (Orbigen) to generate rBVs.

##### Generation of BVM Library

The generation of pMHC display libraries within the baculovirus expression system has been described (41–43). Briefly, DNA from the library acceptor virus, L^d^-eGFP, was isolated and linearized using SceI and CeuI homing enzymes, releasing the eGFP insert. Peptide library fragments were generated by PCR using specific primers that randomize predetermined nucleotides and encode each of the 20 amino acids. The BVM library was designed with the following sequence: SP*X*Y*X*Y*XX*(F/L), where *X* indicates a randomized position. Positions 2 (P2) and 9 (P9) were held constant with proline and phenylalanine/leucine, respectively, because these residues are highly conserved anchor residues for the H-2L^d^ molecule (44, 45). A second PCR was performed using primers that insert compatible cohesive BstXI restriction sites. Ligated BV DNA was transfected into Sf9 insect cells using calcium phosphate. After 3 days, insect cells were screened by flow cytometry for H-2L^d^ and eGFP expression.

##### Enriching BVM Library Using Soluble T Cell Receptors

As described previously (42), the pre-enriched BVM library viruses were used to infect 3 × 10^7^ Sf9 cells at a low multiplicity of infection (MOI = 0.1). After 3 days, the cells were washed with Hanks' balanced salt solution (Mediatech) and stained with soluble fluorescent TCR (100 μg/ml) and an MHC-specific antibody, 28.14.8s (46) (30 μg/ml). This multimer TCR reagent was prepared by incubating Alexa Fluor® 647 Streptavidin conjugates with a high affinity biotinylated anti-mouse Cα mAb, ADO-304 (47). TCR^+^ 28.14.8s^+^ eGFP^−^ insect cells were separated using a MoFlo® high performance cell sorter (Dako-Cytomation), and the isolated cells were added to a fresh flask of 3 × 10^7^ insect cells. This process was repeated until a clear population of TCR^+^ cells was visible by flow cytometry. Individual peptides were identified by single cell-sorting positive cells into 96-well plates containing 10^5^ Sf9 insect cells. Viruses were grown for 7–10 days, and the supernatant was used to infect insect cells, which were stained with TCR and 28.14.8s antibody as above. DNA from TCR^+^ 28.14.8^+^ eGFP^−^ cells was extracted, and the peptide region was PCR-amplified. The PCR products were sequenced, and individual peptides were identified.

##### High Throughput Sequencing of rBV Peptide Libraries

Purified rBVs (2.5 × 10^9^ infectious particles) containing specific peptide libraries were concentrated by ultracentrifugation at 100,000 × *g* for 2 h and resuspended in 50 ml of extraction buffer (10 mm Tris, pH 8.5, 0.01% gelatin, 0.45% Triton X-100, 0.45% Tween 20, 50 mm KCl, 20 units of proteinase K). Viruses were digested for 2 h at 56 °C, and the proteinase K was inactivated for 20 min at 95 °C. A 5-fold excess of template DNA was used to cover the maximum theoretical nucleotide diversity of the library (32^4^ × 2 = 2,097,152 unique nucleotide sequences). PCR amplification from viral DNA was performed using LongAmp Taq (New England Biolabs) and primers containing sequences specific to Illumina flow cells (in italic type), a 6-base index (underlined; CGTGAT, ACATCG, GCCTAA, and TGGTCA) in the forward primer and 8 nucleotides of random sequence preceding the biological binding site (in boldface type) in both primers: forward primer, 5′-*caagcagaagacggcatacgagat*indexx*gtgactggagttcagacgtgtgctcttccgatct*nnnnnnnn**actgaccggcttgtatgct**-3′; reverse primer, 5′-*aatgatacggcgaccaccgagatctacactctttccctacacgacgctcttccgatct*nnnnnnnn**cacttagatggcccgcc**-3′. Products were resolved on a 2.5% low melt agarose gel, and bands of appropriate size were excised and purified. Single-end 100-bp sequencing of the PCR products was performed using an Illumina HiSeq 2500 at the University of Colorado Next-Gen Sequencing Core. Base sequences were interpreted using Illumina's Off-Line Base Calling software due to the homogenous nature of the 5′-end of each amplicon. High quality reads were uploaded to the Galaxy server at Penn State University for sequence grooming, trimming, and analysis of peptide sequences.

##### Vaccines

In general, mice were immunized two times, 7 days apart. Seven days following the second vaccine (day 0), mice were either challenged with CT26 tumor cells or sacrificed. Vaccines were generated by culturing 3 × 10^7^ infected Sf9 insect cells in T175 flasks in 30 ml of Grace's complete medium. To ensure efficient and consistent infections, an MOI of 2 was used to infect plated insect cells, which were then cultured for 3 days. A virus encoding the β-gal peptide was included as a negative control for nonspecific vaccine-induced responses (38). The cells were washed in Hanks' balanced salt solution (Mediatech), and mice were primed by intraperitoneal injection of 5 × 10^6^ or 10^7^ insect cells. Seven days following the prime, mice were boosted with infected insect cells again or 100 μg of the indicated peptide, 50 μg of agonistic anti-CD40 antibody (F6K4.5; BioXcel), and 50 μg of poly(I:C) (Amersham Biosciences) intraperitoneally as described (48).

##### TCR Sequencing and Analysis

High throughput sequencing of AH1-specific CD8^+^ T cells has been described previously (40, 49). Briefly, mice were immunized as above, and in some cases, splenocytes were pooled based on the vaccine. AH1-tet^+^ T cells were sorted, mRNA was isolated using TRIzol®, and first strand cDNA was generated using random hexamers and SuperScript III reverse transcriptase (Invitrogen). High throughput sequencing PCRs were performed as described for all TRBV-13 family members with barcode identifiers for each vaccine type (40). TRAV-6- and TRAV-21-specific primers were included in some experiments. Amplicons of 300–400 bp were quantified by fluorescent measurement using the QubiT® dsDNA HS assay (Invitrogen). Equimolar pools of barcoded amplicons were templates in an emulsion PCR using the 454 GS FLX titanium system (Roche Applied Science) according to the manufacturer's recommendations. Sequences were divided into databases according to the source of AH1-specific T cells. The database was then searched for sequences that matched the CT TCR and 1D4 TCR.

##### Antibodies, Staining Reagents, and Flow Cytometry

Blood lymphocytes were isolated using Ficoll-Paque PLUS (GE Healthcare). Splenocytes were isolated and treated with ammonium chloride-potassium lysis buffer and filtered through a 100-μm cell strainer in complete medium. 1–2 × 10^6^ cells were incubated at room temperature for 90 min with peptide-loaded tetramer (36), FcR Ab (2.4G2), viability-discriminating agent 7-aminoactinomycin D (Sigma), and fluorochrome-conjugated Abs against CD8 (53-6.7, Biolegend), CD11a (M17/4, Biolegend), CD4 (RM4-5, Biolegend), B220 (RA3-6B2, Biolegend), and I-A/I-E (M5/114.15.2, Biolegend). CD4/B220/I-A/I-E Abs and 7-aminoactinomycin D are collectively referred to as the “dump” gate.

Insect cells were cultured at 2 × 10^6^/well in 6-well plates, infected with rBV (MOI = 2), and stained with Abs recognizing H-2L^d^ (28.14.8s), TCRβ (HAM-597), soluble fluorescent TCR molecules, or H-2L^d^ tetramer. Cells were analyzed on a CyAn flow cytometer (Beckman Coulter) or FACSCalibur flow cytometer (BD Biosciences), and data were analyzed using FlowJo software (Tree Star).

##### Intracellular Cytokine Staining

One week following the second vaccination, splenocytes (2 × 10^6^) were stimulated with the indicated peptide and GolgiStop in 96-well plates for 5 h per the manufacturer's instructions (BD Cytofix/Cytoperm Plus fixation/permeabilization kit, BD Biosciences). In some experiments, cell suspensions were incubated with 1 × 10^5^ Sf9.ICAM/B7.1. Cells were stained with surface Abs against CD8, B220, CD4, I-A/I-E, and CD11a. Following fixation and permeabilization, cells were stained with Ab against mouse IFNγ for 1 h at 4 °C. The frequency and number of IFNγ^+^ T cells was determined after subtracting the background staining of β-gal-vaccinated mice stimulated with the same peptide.

##### Tumor Challenge

One week following the second vaccination (day 0), mice were challenged with 5 × 10^4^ CT26 tumor cells subcutaneously in the left hind flank (37). Tumor-free survival was assessed by palpation of the injection site, and mice were sacrificed when the tumors reached 100 mm^2^.

##### Statistical Analyses

Tumor-free survival was analyzed on Kaplan-Meier survival plots, and statistical significance was analyzed with Prism version 4.0 (GraphPad), using the log rank test. All other analyses were performed using an unpaired two-tailed Student's *t* test or, where indicated, a one-way analysis of variance with Tukey's multiple-comparison test. A *p* value of <0.05 was considered statistically significant, and *error bars* represent S.E.

## RESULTS

### 

#### 

##### The 1D4 TCR Is Representative of the AH1-specific T Cell Response

CT, a CD8^+^ AH1-specific T cell clone, was originally identified following limiting dilution and stimulation of spleen cells of BALB/c mice immunized with irradiated CT26 tumor cells expressing GM-CSF (37). Characterization of the TCR from this clone revealed its relatively high affinity (5–6 μm) for the AH1 peptide bound to the H-2L^d^ class I molecule (36, 37). Most tumor-specific TCRs bind with weaker affinity (50, 51). Subsequent evaluation of the *ex vivo* T cell response initiated by mimotope vaccines revealed increased frequency of TRBV-13^+^ T cells, but the CT clone is rarely elicited (40). In fact, mining of over 118,000 TRBV-13 sequences from AH1-specific T cells identified the CDR3β region of the CT clone only six times (Table 1). Perhaps more importantly, the CDR3β was not identified in 48,116 sequences analyzed after vaccination with native AH1 or from unvaccinated tumor-infiltrating lymphocytes. Of note, the CT CDR3α sequence was also not identified in the AH1-specific T cells from tumor-infiltrating lymphocytes or after vaccination.

**TABLE 1:**
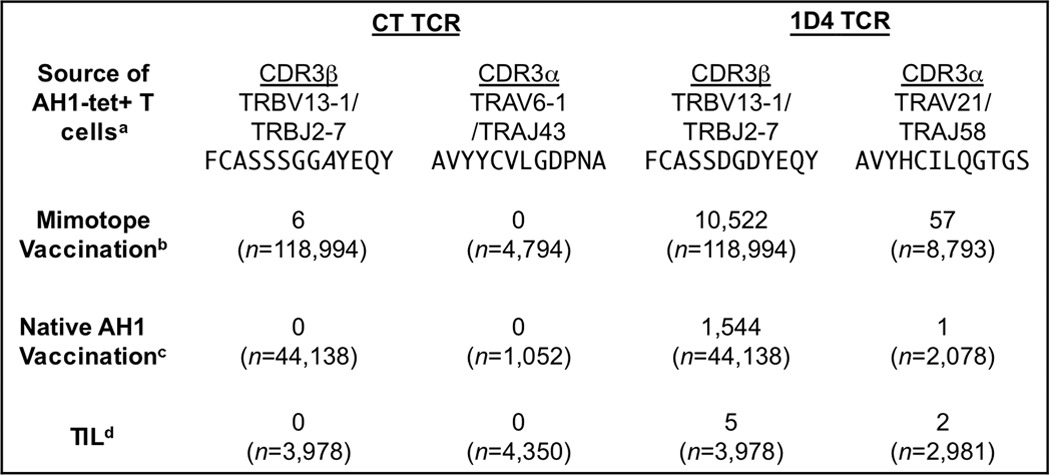
**The 1D4 TCR-Vβ gene fragment is frequently identified in the AH1-specific repertoire**

*^a^* AH1-tet^+^ T cells were FACS-sorted from the indicated sources. Data from 1–3 independent high throughput sequencing runs were compiled and searched for the CT and 1D4 TCR α- and β-chains.

*^b^* AH1-tet^+^ T cells from mice immunized with previously described CT-identified mimotopes (40, 49). *n* varies depending on the experiment and TRBV/TRAV.

*^c^* AH1-tet^+^ T cells from AH1-vaccinated mice (40, 49).

*^d^* TIL, tumor-infiltrating AH1-specific T cells isolated from tumors that had reached ∼100 mm^2^ 12–14 days postinjection.

Sequencing of AH1-specific TCRs revealed a selected motif within the CDR3β region that correlated with tumor protection (40). To investigate the function of this motif, we subcloned the TCR from the 1D4 T cell clone, which encoded the TRAV-21, TRAJ-58, TRBV-13-1, and TRBJ2-7 gene segments (40). The CDR3β region of the 1D4 TCR was identified within AH1-specific tumor-infiltrating lymphocytes, in T cells responding to the AH1 vaccine, and in nearly 8.5% of all TRBV-13^+^ T cells following immunization with mimotopes (Table 1). These results suggest that the 1D4 TCR is more “representative” of the AH1-specific repertoire than the CT TCR. We used this clone to test the hypothesis that screening mimotope libraries with T cells that are representative of endogenous T cells improves the discovery of efficacious mimotopes.

##### 1D4 TCR Has Low Affinity for the Self-antigen, AH1·H-2L^d^

**FIGURE 1.**
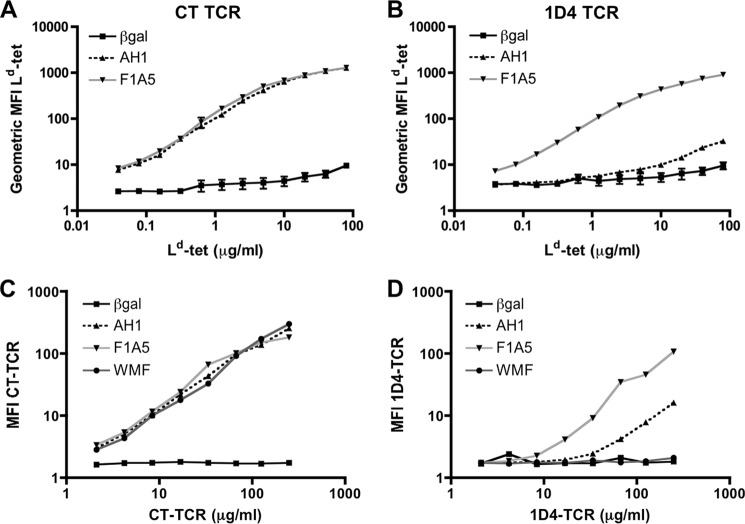
**The CT TCR binds AH1·H-2L^d^ with increased affinity relative to the 1D4 TCR.** Sf9 insect cells were infected with recombinant BV expressing transmembrane CT TCR (*A*) or 1D4 TCR (*B*). Three days postinfection, the cells were stained with increasing concentrations of H-2L^d^ tetramer loaded with the indicated peptide and TCRβ-specific antibody (clone HAM-597). The geometric MFI of tetramer staining was normalized to TCR expression and plotted. Combined data from three independent experiments are shown. *C* and *D*, insect cells were infected with recombinant BV expressing transmembrane pMHC, as indicated and stained with soluble CT TCR (*C*) or 1D4 TCR (*D*) and an MHC-specific antibody (clone 28.14.8s). Geometric MFI of TCR staining is shown after normalizing for MHC expression levels (38). Data shown are representative of three independent experiments with similar results.

To determine the relative binding of the AH1·H-2L^d^ complex to the CT and 1D4 TCRs, we performed flow cytometry-based binding experiments in which the staining intensity correlates with the monomeric binding affinity of the TCR to the pMHC (38, 42). We produced soluble and transmembrane-bound TCR and pMHC molecules expressed by rBV-infected insect cells. Following infection of insect cells with rBV-expressing transmembrane molecules, we stained the cells with increasing concentrations of H-2L^d^ tetramer (Fig. 1, *A* and *B*) or soluble TCR molecules (Fig. 1, *C* and *D*), respectively. In addition to the AH1 peptide, binding of one protective mimotope (F1A5) and one non-protective mimotope (WMF), each identified by the CT TCR, was also examined. The CT TCR bound to AH1, F1A5, and WMF complexed to H-2L^d^ molecules similarly (Fig. 1, *A* and *C*). This result is inconsistent with previous experiments, showing that the F1A5·H-2L^d^ complex bound CT TCR with higher affinity than the AH1·H-2L^d^ complex (39). The explanation for this inconsistency is that we inserted cysteines in the linker between β_2_-microglobulin and the peptide and the MHC molecule, which form a disulfide bond (52), trapping the peptide in the MHC groove. This made it possible to detect the low affinity interaction between the 1D4 TCR and AH1·H-2L^d^ in this assay. This “disulfide trap” maximized CT TCR binding and prevented the detection of affinity differences with this TCR.

The 1D4 TCR bound to F1A5 with higher intensity than AH1 in both assays (Fig. 1, *B* and *D*). These results suggest that the affinity of the 1D4 TCR for the native AH1 antigen is lower than that of the CT TCR. Interestingly, the 1D4 TCR did not bind the non-protective WMF mimotope at any concentration, suggesting that the 1D4 TCR discriminates between these mimotopes, unlike the CT TCR. Despite having a lower affinity for the AH1·H-2L^d^ antigen, the increased representation within the AH1-specific repertoire and lack of recognition of the non-protective WMF mimotope suggested that the 1D4 TCR may identify different, potentially protective mimotope vaccines.

##### The BVM Peptide Library Is Diverse

**FIGURE 2.**
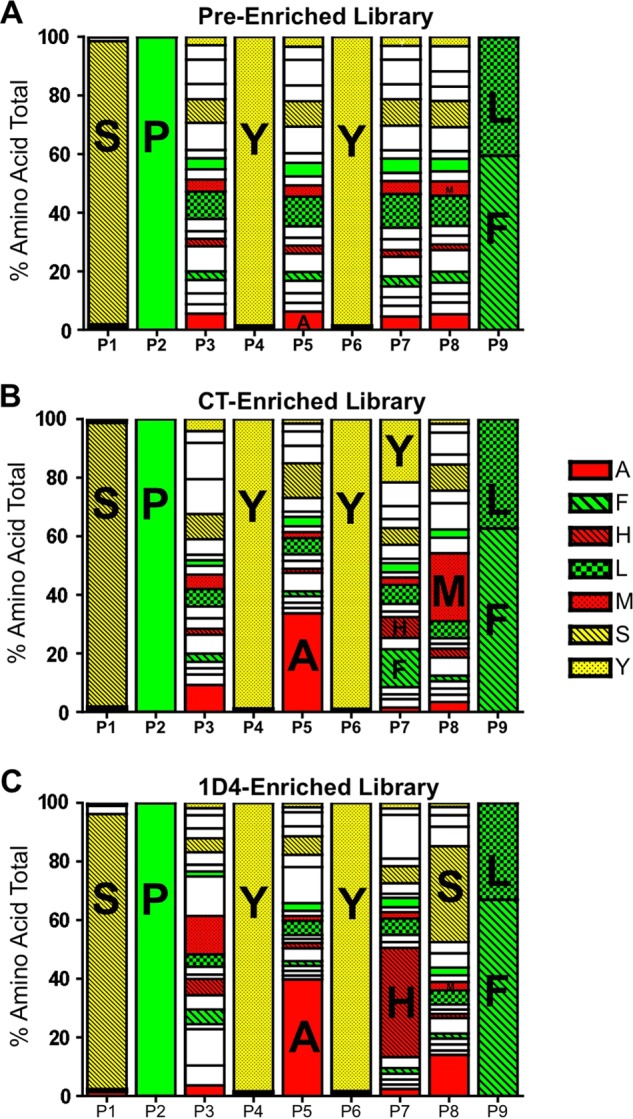
**The CT and 1D4 TCRs enriched unique amino acids from the diverse peptide library.** Baculovirus DNA from the pre-enriched BVM library (*A*), CT-enriched library (*B*), and 1D4-enriched library (*C*) was sequenced using the Illumina Hi-Seq2500 platform. Sequences were aligned with the oligonucleotide used to generate the BVM library that encoded the proline at P2 and phenylalanine/leucine at P9. Amino acids of particular interest are indicated by *one-letter code* and *color*.

To determine whether the CT or 1D4 TCR identified peptides that differentially protected against tumor growth, we generated a peptide library (BVM library) to screen for potential mimotopes using each TCR. We inserted a PCR fragment that encoded peptides with degenerate nucleotides in strategic positions into the BV genome (42). The library peptides were linked to β_2_-microglobulin and co-expressed with the H-2L^d^ heavy chain. The amino acids within the AH1 peptide predicted to form secondary interactions with the MHC molecule were randomized: SP*X*Y*X*Y*XX*(F/L) (where *X* is any amino acid). To estimate the size and determine the overall composition of the BVM library, we performed high throughput sequencing using the Illumina Hi-Seq2500 platform (Table 2). If all possible amino acid combinations were represented in the library, then we would expect 388,962 unique peptide sequences (21^4^ × 2, includes stop codons). When we analyzed the sequences that adhered strictly to the library design, we identified over 297,000 unique peptides (Table 2). When unique sequences identified less than three times were eliminated from the analysis, the number of unique peptides dropped to 180,000. However, we also analyzed the sequences using more lenient criteria that encoded a proline at P2 and a phenylalanine or leucine at P9 (XP*XXXXXX*(F/L)) and identified over 734,000 unique sequences (268,000, identified ≥3 times). Approximately 5.8% of all nucleotide sequences could be attributed to PCR errors and oligonucleotide synthesis errors, as determined by changes in the oligonucleotide sequence used to build the library. Thus, we estimated that the library was ∼70% saturated, and all 20 amino acids were represented and well balanced (Fig. 2*A*).

**TABLE 2:**
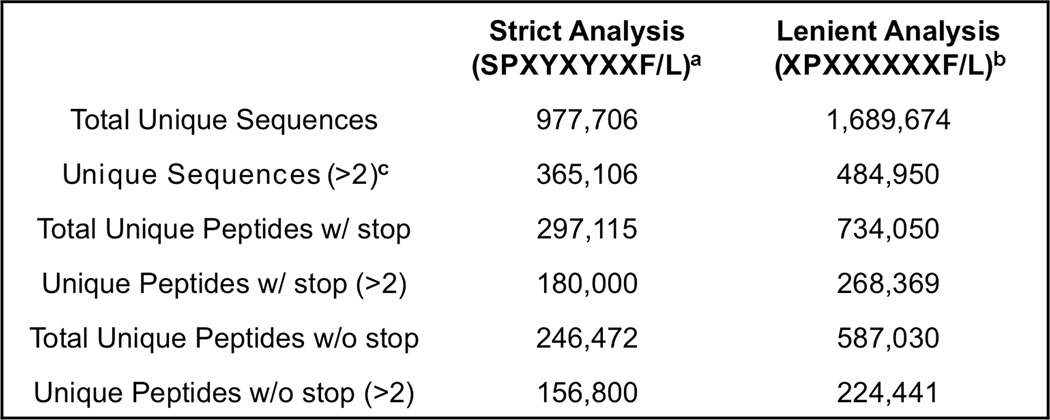
**Sequencing of the pre-enriched BVM library** The pre-enriched BVM library contained 62,688,573 total sequences.

*^a^* Library primer search parameters: TCC CTT NNS TAT NNS TAC NNS NNS YTY.

*^b^* Search parameters were focused on DNA sequences that contained the MHC anchor amino acids of proline at P2 and F/L at P9: NNN TCC NNN NNN NNN NNN NNN NNN YTY.

*^c^* Only sequences found three times or more were included in the analysis.

##### The CT and 1D4 TCRs Enrich Different Peptides from the BVM Library

After infection of the insect cells with the BVM library, the pMHC molecules traffic to the cell membrane and are available for binding by fluorescently labeled multimerized TCR molecules. The BVM library was enriched using soluble 1D4 or CT TCR molecules and fluorescence-activated cell sorting (42). The library was enriched three times by the CT TCR and four times by the 1D4 TCR due to low TCR staining intensity. The libraries were sequenced postenrichment and their amino acid composition was analyzed using the lenient motif (*X*P*XXXXXX*(F/L)) (Fig. 2, *B* and *C*). Many peptides identified using the CT TCR contained an alanine at P5 and a large, uncharged, aromatic amino acid (Trp, Phe, or Tyr) at P7 (Fig. 2*B*). Alanine at P5 of the AH1 peptide was previously shown to be preferred for recognition by the CT TCR, and a single substitution at this position in the AH1 peptide is protective (37). The 1D4-enriched library also preferentially selected an alanine at P5 (Fig. 2*C*). However, histidine, the natural amino acid at position 7 in the AH1 peptide, was noticeably enriched in peptides identified by the 1D4 TCR. We detected subtle differences at P8 within each enriched library; the CT-enriched library was dominated by a methionine, whereas the 1D4-enriched library was dominated by serine. These results suggest that each AH1-specific TCR selects different peptides from the library.

##### Most Peptides Enriched by the CT TCR Do Not Bind the 1D4 TCR

**FIGURE 3.**
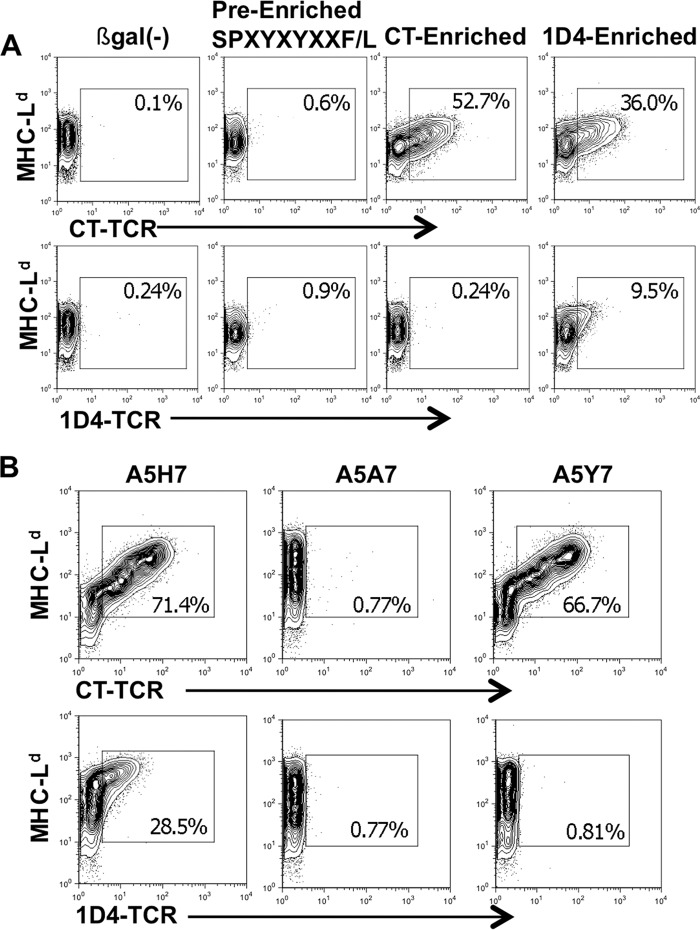
**Most peptides enriched from the BVM library using the CT TCR do not cross-react with the 1D4 TCR.** *A*, Sf9 insect cells were infected with the BVM library viruses, and cells that bound soluble TCR were enriched by FACS sorting, and the process was repeated. Shown are fresh insect cells infected with TCR-enriched viruses stained with fluorescently labeled TCR and an MHC-specific antibody and their frequency. Plots shown are representative of three independent experiments with similar results. *B*, Sf9 insect cells were infected with the indicated pMHC virus stained 3 days later with either the soluble CT TCR (*top*) or 1D4 TCR (*bottom*). *Numbers* reflect the frequencies of MHC^+^ TCR^+^ cells within the live cell gate. Plots shown represent three independent experiments.

We next tested whether the 1D4 TCR cross-reacted with the mimotopes identified from the library by the CT TCR. We infected insect cells with the CT-enriched peptide library and stained them with either the CT or 1D4 TCR (Fig. 3*A*). Similar to the WMF peptide, the 1D4 TCR did not bind the CT-enriched peptide library, suggesting that the 1D4 TCR does not recognize the dominant CT binding peptides enriched from this library. Conversely, peptides enriched with 1D4 TCR cross-react with CT TCR, suggesting that CT TCR could identify these mimotopes.

Next we determined the significance of the P7 amino acid for recognition by both TCRs. We generated an rBV that expressed the protective A5 peptide, which naturally contains a histidine, and mutated P7 to an alanine or tyrosine. The mutant viruses were then used to infect insect cells and stained with either CT TCR or 1D4 TCR (Fig. 3*B*). Not surprisingly, the alanine substitution at P7 (A5A7) abrogated TCR recognition by both TCRs. The tyrosine substitution (A5Y7) did not affect CT TCR recognition, as predicted by similar peptides identified from the library using this TCR. However, 1D4 TCR recognition of the A5Y7 peptide was ablated, implicating the histidine at P7 as an important residue for 1D4 TCR recognition.

##### Enriched Libraries Contain Different Peptides

To characterize the peptides of the TCR-enriched libraries, we sequenced them using the Illumina HiSeq platform and cloned individual BVs. The most frequently identified peptides were enriched up to 440-fold relative to the pre-enriched library (Table 3). The top 10 most frequent sequences identified by the CT TCR account for almost 20% of the enriched library, whereas those identified by the 1D4 TCR account for almost half of the enriched library. This difference may be accounted for by the diverse range of peptides that bind to the CT TCR or the additional sort performed with the 1D4 TCR. This sort was performed because the intensity of the 1D4 staining was significantly less than the CT TCR staining (Fig. 3*A*), suggesting a less efficient sort or lower affinity peptides.

**TABLE 3:**
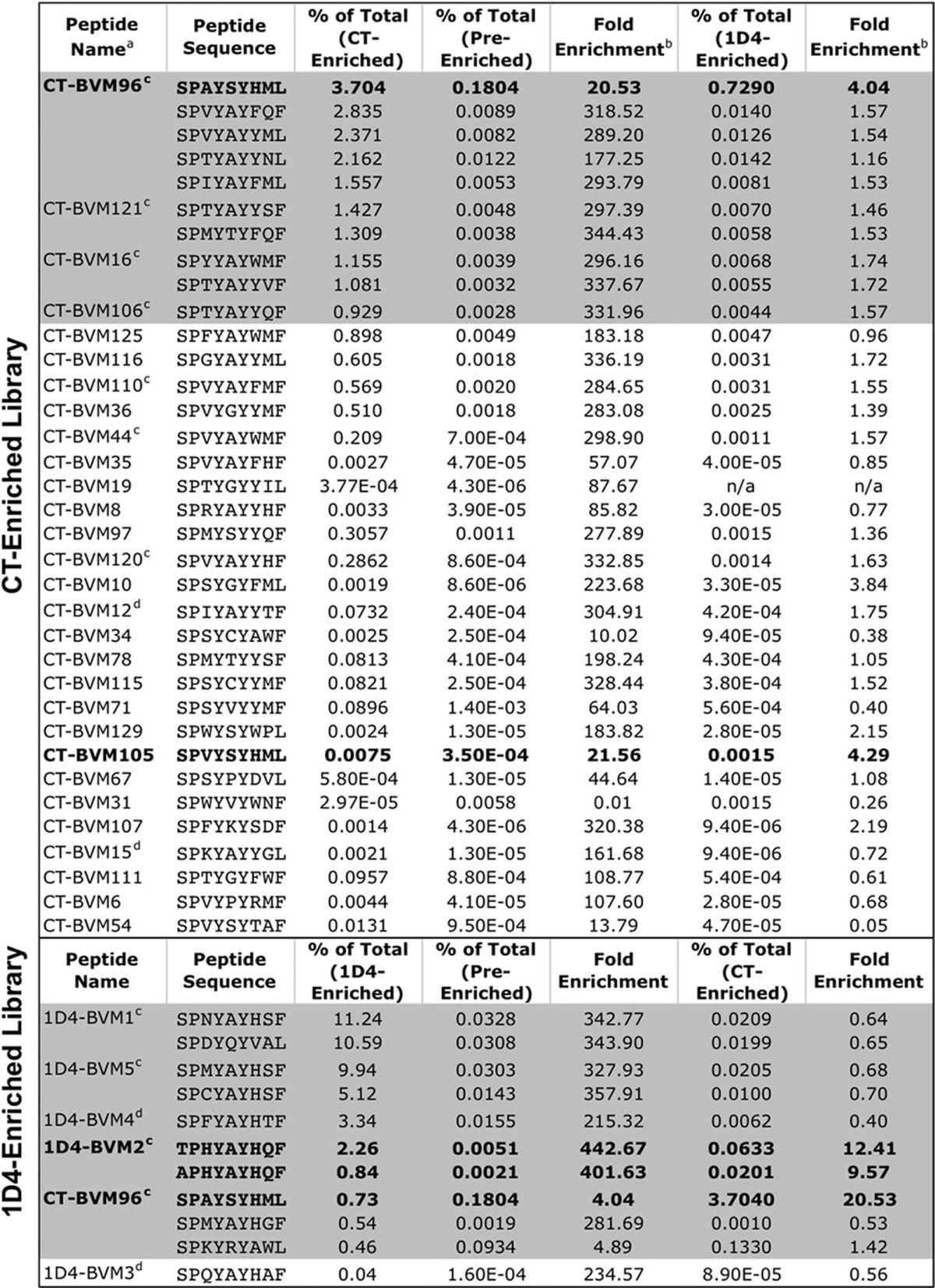
**Multiple mimotopes from the BVM library screened by CT and 1D4 TCRs were identified and cloned** Gray shading indicates the 10 most frequently identified peptides from each library. Boldface type indicates mimotopes that were enriched >4-fold using both TCRs.

*^a^* Peptide names were assigned to any pMHC-encoding virus that was cloned from a TCR-enriched library.

*^b^* Determined by dividing the frequency of the indicated peptide within the TCR-enriched library by the original frequency within the pre-enriched library.

*^c^* Mimotopes tested for AH1-specific T cell expansion, cytokine production, and tumor protection.

*^d^* Mimotopes tested for tumor protection only.

Consistent with Fig. 3*A*, the top peptides enriched by 1D4 TCR were not enriched by CT TCR. However, the most frequently identified peptide by the CT TCR (CT-BVM96) was also found in the 1D4-enriched library. Importantly, CT-BVM96 was highly represented in the pre-enriched library, which may contribute to its high representation in both libraries. 29 peptides were cloned from the CT-enriched library, and five peptides were cloned from the 1D4-enriched library for further characterization. Several of these peptides were also the most frequently identified by sequencing. Overall, individually cloned peptides from each sorted library were significantly enriched and dominant within the postenrichment libraries.

##### Increased Number of AH1-specific T Cells following Immunization with Individual Mimotopes Identified by the 1D4 TCR

**FIGURE 4.**
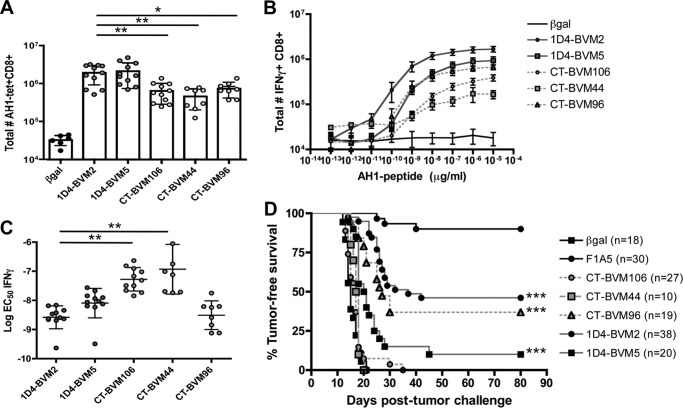
**Individual mimotopes identified from the BVM library elicit AH1-specific T cells that make IFNγ and vary in anti-tumor immunity.** Mice were immunized two times 7 days apart with Sf9 insect cells infected with recombinant BV encoding single mimotopes cloned from the 1D4-enriched or CT-enriched library. *A*, the total number of AH1-tet^+^ CD8^+^ cells in the spleen was determined 7 days following the second vaccine. Data shown are combined from three independent experiments (*n* = 2–4 mice/group) and compared using a one-way analysis of variance with Tukey's multiple-comparison test (**, *p* < 0.01; *, *p* < 0.05). *B*, splenocytes were stimulated with increasing concentrations of AH1 peptide, and the total numbers of IFNγ^+^ CD8^+^ T cells were plotted. Data shown are combined from three independent experiments, *n* = 2–4 mice/group. *C*, the percentage maximum of IFNγ for each mouse from *B* was plotted, and individual peptide dose-response curves were generated. The EC_50_ value for IFNγ production was determined by fitting the data to a variable slope sigmoidal dose-response curve using GraphPad Prism software. Data shown are from three independent experiments combined, *n* = 2–4 mice/group. *D*, immunized mice were challenged with 5 × 10^4^ CT26 tumor cells 7 days following the boost vaccine (day 0). Tumor growth was monitored for 80 days and sacrificed when tumors reached 100 mm^2^. The groups indicated were compared with the negative control (β*gal*) using the log rank test and GraphPad Prism version 4.0 software. *Error bars*, S.E.

We previously demonstrated that immunization with insect cells infected with rBV-expressing pMHC induces a robust antigen-specific immune response to the recombinant peptide (38). In a pilot study using this vaccine strategy, we screened mice for AH1-specific T cells responding to 34 cloned rBVs. We chose three peptides from the CT-enriched library and two peptides from the 1D4-enriched library for further characterization. These peptides either elicited the most AH1-specific T cells, were identified the most frequently, or contain the preferred amino acid at P7 (data not shown). Mice were immunized as described, and splenocytes were harvested and analyzed for AH1-specific responses 7 days following the second vaccine. Both peptides from the 1D4-enriched library (1D4-BVM2 and 1D4-BVM5) elicited more AH1-specific T cells compared with the CT-enriched peptides (Fig. 4*A*). Responses to the peptides identified by 1D4 and CT were stronger than the response to the negative control vaccine (β-gal), which included endogenous AH1-specific T cells and background staining of the AH1-tetramer. When stimulated with AH1 peptide *ex vivo*, more T cells elicited by the 1D4-BVM2 mimotope produced IFNγ at most concentrations of peptide (Fig. 4*B*). There was little difference between the 1D4-BVM5 mimotope and CT-BVM96, whereas CT-BVM44 and CT-BVM106 produced significantly less IFNγ (Fig. 4*B*). Further characterization of IFNγ production at all peptide concentrations revealed distinct differences in the functional avidity of the T cells, as determined by the EC_50_ concentration (Fig. 4*C*). 1D4-BVM2 and CT-BVM96 had lower EC_50_ concentrations, suggesting increased functional avidity for the AH1 antigen. CT-BVM44 and CT-BVM106 had significantly higher EC_50_ concentrations, indicative of a lower avidity T cell population.

Mice immunized with 1D4-BVM2, 1D4-BVM5, and CT-BVM96 were significantly protected from subsequent CT26 tumor challenge when compared with the negative control (Fig. 4*D*). The protection by CT-BVM96 was not unexpected due to the presence of histidine at P7, similar to mimotopes frequently identified by the 1D4 TCR. Additionally, this mimotope weakly cross-reacted with the 1D4 TCR (data not shown) and was enriched slightly using the 1D4 TCR (Table 3). We screened several other CT-enriched mimotopes for tumor protection and did not observe any significant protection (Table 3). Although not all 1D4-enriched mimotopes were protective, these results suggested that the representative TCR was selecting a better pool of mimotopes and led us to test whether these pools of enriched peptides could be used in vaccines.

##### Immunization with a Pooled Mixture of Mimotopes Enriched by 1D4 TCR Results in High Quality T Cell Responses

**FIGURE 5.**
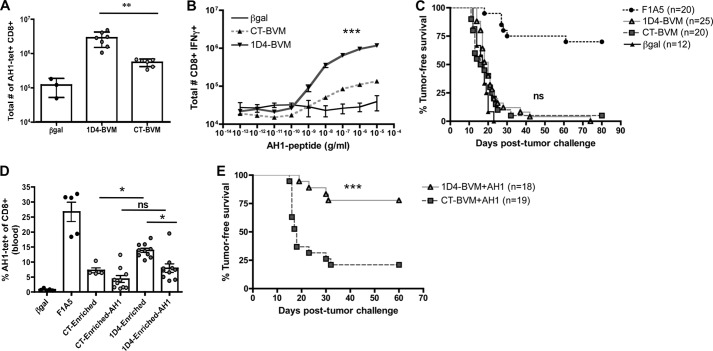
**Immunization with the pool of 1D4-enriched peptides elicits AH1-specific cells and improves anti-tumor immunity when boosted with native tumor antigen.** Mice were immunized with Sf9 insect cells infected with the pool of peptides from the BVM library enriched by 1D4 and CT TCRs. *A*, the total number of AH1-tet^+^ CD8^+^ cells in the spleen was determined 7 days following the boost vaccine. Data shown are combined from two independent experiments with *n* = 2–3 mice/group and compared using a Student's *t* test (**, *p* = 0.0017). *B*, splenocytes from *A* were stimulated with increasing concentrations of AH1 peptide, and the total number of IFNγ^+^ CD8^+^ T cells was determined and plotted. Data shown are combined from two independent experiments with *n* = 2–3 mice/group and compared using Student's *t* test (***, *p* < 0.0001). *C*, mice were immunized as above and challenged with 5 × 10^4^ CT26 tumor cells 7 days following the boost vaccine. Tumors were measured for 80 days and sacrificed when tumors reached 100 mm^2^. *D*, mice were primed as in *C* and then either boosted with the same mimotope vaccine or with 100 mg of native AH1 peptide in combination with 50 mg of poly(I:C) and 50 mg of anti-CD40 antibody (49). Peripheral blood mononuclear cells were analyzed 7 days following the boost on the day of tumor challenge (day 0). The frequency of AH1-tet^+^ CD8^+^ T cells was plotted. These results are from one of two independent experiments with 5–10 mice/group. Data were analyzed using one-way analysis of variance with Tukey's multiple-comparison test (*, *p* < 0.05). *E*, mice were primed with the bulk enriched peptide library and boosted with the native AH1 peptide as in *D* and challenged with CT26 tumor 7 days following the boost. Mice were monitored for tumor growth and sacrificed when the tumors reached 100 mm^2^. Survival curves were analyzed using the log rank test and GraphPad Prism version 4.0 software (***, *p* < 0.0001). *Error bars*, S.E.

We hypothesized that mimotope mixtures would stimulate a more broadly cross-reactive T cell population, thereby improving anti-tumor immunity. We immunized mice with insect cells infected with each of the enriched libraries and examined the AH1-specific T cell responses in the peripheral blood and spleen. As in Fig. 4, which analyzed individual mimotopes, vaccination with the 1D4 TCR-enriched library elicited more AH1-tet^+^ CD8^+^ T cells in the spleen and a greater number of CD8^+^ T cells that produce IFNγ upon stimulation with AH1 peptide *ex vivo* (Fig. 5, *A* and *B*). Despite this difference in cell number, we observed no difference in tumor protection between the two vaccine cohorts (Fig. 5*C*).

We previously reported that immunization with mimotope vaccines is dramatically improved by boosting the initial mimotope response with the native tumor antigen (49). We demonstrated that boosting with the native peptide, AH1, enhanced the expansion of high affinity tumor-specific T cells, and it may also limit the expansion of non-cross-reactive or low affinity T cells. We next asked whether boosting with the native AH1 peptide would improve the immune response elicited by the 1D4-enriched library vaccine compared with the CT-enriched vaccine. Mice were immunized with either the CT or 1D4-enriched libraries and boosted with either another dose of the library vaccine or the native AH1 peptide. Consistent with our previous results, we observed a decrease in the overall frequency of AH1-tet^+^ CD8^+^ cells in the blood following the AH1 boost and no difference in cell frequency between mice immunized with 1D4-enriched library or the CT-enriched library (Fig. 5*D*). However, following tumor challenge, mice immunized with the 1D4-enriched library and boosted with the AH1 peptide had significantly enhanced tumor-free survival relative to the CT-enriched library boosted with AH1 (Fig. 5*E*). These results confirm that the 1D4 TCR selects mimotopes that expand a greater number of AH1-specific T cells, and these cells improve anti-tumor immunity when subsequently focused with the native tumor antigen.

##### 1D4-enriched Mimotopes Cross-react with a Protective T Cell Repertoire

**FIGURE 6.**
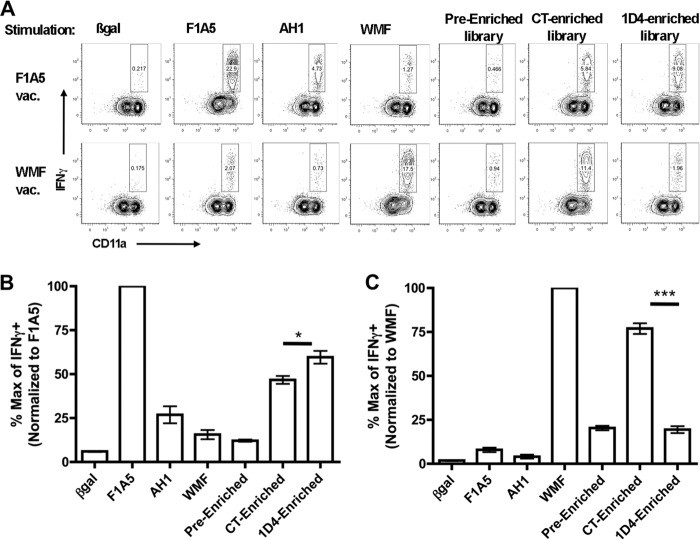
**Peptides enriched by the 1D4 TCR stimulate T cells elicited by protective mimotope vaccines more effectively than peptides enriched by the CT TCR.** *A*, mice were immunized with either the protective F1A5 mimotope or the non-protective WMF mimotope. 2 × 10^6^ splenocytes were isolated 7 days following the boost and incubated with 1 × 10^5^ insect cells that express ICAM and B7 co-stimulator molecules and were infected for 3 days with rBV that encoded the indicated pMHC. T cells were stimulated in the presence of monensin for 5 h and stained with surface antibodies and intracellular IFNγ. Plots shown are gated on CD8^+^ CD4/B220/MHCII^−^ T cells. Representative plots from one of three independent experiments are shown. Splenocytes from mice immunized with F1A5 (*B*) or WMF (*C*) were stimulated with the pMHC indicated. The total frequency of IFNγ^+^ cells/mouse was normalized to the maximum value from the immunizing mimotope (F1A5 or WMF), and all other percentages were compared with that maximum. The groups shown were compared using Student's *t* test (*, *p* = 0.038; ***, *p* < 0.0001). *Error bars*, S.E.

We hypothesized that peptides enriched by the 1D4 TCR stimulate T cells elicited by a protective mimotope vaccine more effectively than peptides enriched by the CT TCR. To determine if the 1D4-enriched library cross-reacts with a known protective repertoire, we immunized mice with a previously described mimotope, F1A5, which elicits a high frequency of T cells that express the characterized CDR3β motif and protects mice from tumor challenge (40). Another group of mice was immunized with the non-protective WMF peptide, which elicits AH1-specific T cells with a low frequency of motif-expressing TCRs that do not protect mice from tumor challenge. Splenocytes from immunized mice were stimulated with insect cells infected with rBVs expressing control peptides or the enriched libraries, and IFNγ production was determined by intracellular cytokine staining. The 1D4-enriched library stimulated more robust responses from mice immunized with the protective F1A5 mimotope (Fig. 6, *A* and *B*). Conversely, the CT-enriched library stimulated the non-protective WMF-immunized splenocytes more robustly (Fig. 6, *A* and *C*). These results confirm that the 1D4 TCR primarily selects mimotopes that stimulate a similar repertoire as a known protective mimotope and fails to cross-react with a known non-protective repertoire. We therefore conclude that a more “representative” TCR, even one of lower affinity, may be utilized more effectively in the screening process for mimotope vaccine candidates.

## DISCUSSION

We asked here whether the CT T cell clone was appropriate for effective mimotope selection because it has high affinity for the dominant tumor antigen and effectively kills CT26 tumor cells (37). High affinity T cells are the most logical choice for cancer immunotherapy and antigen selection because they are associated with enhanced effector functions as well as improved antiviral and anti-tumor immunity (53–55). However, central tolerance limits the precursor frequency of T cells expressing TCRs with high affinity for self-tumor antigens, making vaccines that specifically target these cells less effective. We proposed that ideal mimotope vaccines are more frequently identified by representative TCRs rather than by rare high affinity T cell clonotypes, such as the CT TCR.

The 1D4 T cell clone exemplifies a more representative T cell clone. This clonotype expresses the dominant TRBV-13/TRBJ-2.7 TCR and the CDR3β motif, and the sequence of this TCR was identified in naturally responding tumor-infiltrating lymphocytes and vaccine-elicited T cells (Table 1). Consistent with our hypothesis, the 1D4 TCR bound protective mimotopes, such as the F1A5 mimotope, but did not recognize several less protective mimotopes, including the WMF peptide that was identified using the CT TCR (38) (Fig. 1). This specificity suggested that the 1D4 TCR would more efficiently enrich protective peptides from a library. Indeed, high throughput sequencing analysis of the BVM library enriched with the 1D4 TCR identified unique mimotopes that were not identified previously using the CT TCR (Table 3).

The sequencing results showed a difference in peptide diversity between the two enriched libraries; the CT TCR-enriched library was more complex (Table 3). We hypothesize that this complexity is the result of the higher affinity of CT TCR for the self-antigen AH1. It has been reported that high affinity TCRs are more prone to cross-reactivity and deletion during negative selection (56, 57). Although the diversity of the CT TCR-enriched library may be higher, it is unlikely that this is the sole reason for poor anti-tumor immunity following immunization with the bulk library because individual mimotopes tested from this library are also non-protective (Fig. 4).

Most of the peptides identified by the 1D4 TCR contained a histidine residue at P7, similar to the native AH1 peptide. Furthermore, mutations at the P7 histidine abrogated 1D4 TCR binding (Fig. 3*B*), suggesting that TCR binding is focused within this region of the peptide. Histidine, a large basic amino acid, is often protonated at physiologic pH and has a net positive charge. We speculate that acidic residues in the CDR3β motif (glutamic acid and aspartic acid) play a role in binding to the positively charged region of mimotopes enriched by the 1D4 TCR. In contrast, the CT TCR preferentially bound peptides with large polar (tyrosine) or uncharged aromatic residues at P7 (tryptophan and phenylalanine) and frequently selected methionine at P8 (Fig. 2*B*). The additional amino acid and the properties of glycine and alanine within the CDR3β region of the CT TCR may also contribute to greater flexibility, allowing this TCR to adopt different conformations and bind to large aromatic residues, such as tryptophan. Finally, the CT TCR and 1D4 TCR express different α-chains, which probably contribute to their differential peptide selection.

Although the 1D4 TCR has a relatively low affinity for AH1 (Fig. 1), vaccination with mimotopes identified using the 1D4 TCR resulted in increased number and quality of AH1-specific T cells (Figs. 4 and 5). The successful enhancement of tumor protection by boosting with the native AH1 peptide suggests that the peptides enriched by the 1D4 TCR did not elicit significant numbers of T cells with little or low reactivity toward the native tumor antigen. Correlatively, immunization with the CT TCR-enriched peptides may have elicited non-cross-reactive T cells that are not successfully stimulated by the AH1 boost. Although we show a clear difference in anti-tumor immunity between mimotopes identified with each TCR, we did not definitively demonstrate that the representation of each TCR clonotype is the reason for this phenomenon. Other factors, such as the presence of competing mimotope-specific cells, may also impact tumor-specific T cell expansion. Competition between T cells for access to antigen-presenting cells is well documented (58, 59). We are currently exploring how mimotope-specific cells could negatively impact the expansion or function of tumor-specific cells in this system. However, here we demonstrated that 1D4-enriched mimotopes stimulated a “1D4-like” repertoire of T cells, whereas the CT-enriched library stimulated a different repertoire, similar to those elicited by the less protective mimotopes (Fig. 6) (40).

To overcome the unpredictability of pMHC/TCR interactions, recent methods for the identification of mimotopes utilize binding and stimulation of tumor antigen-specific T cells to select vaccine candidates (24, 25, 37). A recent method for identification of mimotopes, called TCR-optimized peptide skewing of the repertoire of T cells (TOPSORT), proposes that effective mimotopes stimulate T cell clones with high affinity but not those with low affinity for the native TAA (9, 25). The components used to develop that model were the opposite of those that we used here. Using a combinatorial peptide library, they identified a mimotope that stimulated robust responses from a high affinity Melan-A/MART-1-specific T cell clone, MEL5, but not from the low affinity MEL187.c5 clone. Rather than high and low affinity T cells, the question we addressed originated from a cognate panel of mimotopes with a range of anti-tumor activity. The new mimotope identified by Ekeruche-Makinde *et al.* (25) elicited a greater frequency of T cells specific for the native epitope in healthy peripheral blood mononuclear cells than a previously identified, less optimal mimotope. Their results demonstrate the potential for using a high affinity T cell clone specific for a TAA to identify mimotopes that enhance anti-tumor T cell responses, although the clonotype must be present to be activated by the mimotope. The authors stated that the MEL5 TCR Vβ-chain is not expressed in all donors, which may explain why 40% of donors did not respond to mimotope stimulation. The overall representation of the MEL5 clonotype in the naturally responding anti-tumor repertoire of individual patients is not known (9, 25). If a dominant clone common to all of the patients could be identified and were used to screen for mimotopes, it may be more consistently effective.

Thus, a TCR clonotype with low affinity for the native TAA may be superior for mimotope selection if the TCR is well represented in the endogenous repertoire of responding T cells. The most efficacious mimotope in this study, 1D4-BVM2, binds to and is enriched by both the 1D4 TCR and CT TCR (Table 3), suggesting that the most effective mimotopes might stimulate lower affinity yet more representative T cell clonotype(s) in addition to rare high affinity T cell clonotype(s). The future of mimotope identification using T cells may therefore rely on characterizing the antigen-specific T cells naturally responding to tumors or public TCRs specific for tumor antigens, thus facilitating the identification of mimotopes that target T cells in the periphery and tumor as well as between different patients. Tumor-specific T cell clones with increased representation or higher precursor frequencies may be the ideal targets for immunotherapy. Ideally, those T cells would also express high affinity TCRs for the tumor antigen, although we argue that this is not a prerequisite for success. Utilizing new high throughput sequencing techniques, it may be possible to characterize and screen tumor-specific TCRs to use for the identification of mimotopes that will enhance the activation of both representative and high affinity T cells.

## Footnotes

3 The abbreviations used are:

TCR: T cell receptor
CDR3: complementarity-determining region 3
pMHC: peptide-MHC
BV: baculovirus
rBV: recombinant BV
eGFP: enhanced GFP
MOI: multiplicity of infection
Ab: antibody.
